# Towards a WHO classification of genetic tumour syndromes

**DOI:** 10.1515/medgen-2025-2029

**Published:** 2025-11-08

**Authors:** Reiner Siebert, William D. Foulkes

**Affiliations:** Ulm University & Ulm University Medical Centre Institute of Human Genetics Ulm Germany; McGill University Department of Human Genetics Montreal Canada

**Keywords:** World Health Organization (WHO), Genetic Tumour Syndromes, Cancer Predisposition, Genes, International Agency for Research on Cancer (IARC)

## Abstract

Almost 70 years ago, the World Health Organization (WHO) decided to propose a “Classification of Tumours”. Since then, a systematic and extensive classification system for tumours has been continuously developed in successive editions and nowadays closely interlinks with coding systems for cancer registries like the International Classification of Diseases for Oncology (ICD-O). Whereas past editions had their focus on histopathological aspects of tumour classification in different organ systems and topologies, to which (somatic) genetic alterations increasingly contributed, the current fifth edition of the WHO Classification for the first time includes a separate “Blue Book” volume on “Genetic Tumour Syndromes”. Along with chapters dedicated to tumour predisposition inferred by constitutional (germline) genetic pathogenic variants in the different organ-specific volumes of the classification, this new addition to the WHO classification highlights the increasing importance of constitutional genetic alterations for the diagnosis and clinical management of patients with such tumours. The WHO classification of Genetic Tumour Syndromes applies a hierarchical system based on four levels: the major (cellular) mechanism affected, the molecular pathway involved, the (clinical) syndrome, and the specific gene(s) affected. It provides – in part novel or modified – names to the genetic tumour syndromes as well as definitions and descriptions of clinical, epidemiologic, etiologic, pathogenetic and pathological aspects. Essential and desirable diagnostic criteria are given as well as rules for reporting, thus paving the way to international standardization. While the final version of the WHO Classification of Genetic Tumour Syndromes is in proof-stage, the present article, which is based on its beta-version, aims to provide an overview of the concepts underpinning the classification.

## Introduction

With the final volume of the fifth edition of the World Health Organization (WHO) Classification of Tumours the International Agency for the Research on Cancer (IARC) presents for the first time a taxonomy of genetic tumour (predisposition) syndromes that provides definitions and sets international standards for their diagnosis. While the Blue Book on “Genetic Tumour Syndromes (GTS)” detailing the classification is in proof-stage, the aim of this article is to provide the reader with key principles underlying the classification along with some information on its development and structure [1, 2].

## WHO classification of tumours – the historical perspective

Almost 70 years ago, back in 1956, a resolution of the WHO Executive Board was the starting point for the development of the “WHO Classification of Tumours” [3]. Since the publication of the first edition in 1967–1981, which essentially listed diagnostic terms with the corresponding International Classification of Diseases for Oncology (ICD-O) morphology codes along with short diagnostic criteria, the form and content of the WHO Classification has continuously evolved [3]. By the third edition which launched in 2000, the IARC took over the task to develop the WHO Classifications and publish the respective editions in the form of so-called “Blue Books”, i. e. organ or topically focused, well-illustrated books containing concise but factually rich descriptions of the epidemiological, morphological, immunohistochemical and clinical aspects of all accepted and provisional tumour entities [4]. The initially predominating histopathologic aspects were gradually supplemented by (mostly somatic) genetic diagnostic criteria highlighting the increasing importance of genetic information, with some tumours defined solely and explicitly by specific genetic alterations. In this regard, the importance of genetic alterations in the WHO Classification of Haematolymphoid Tumours was recently highlighted in Medizinische Genetik [5].

## The 5^th^ edition of the WHO classification of tumours

At the time of writing of this article, the publication of the fifth edition of the WHO Classification of Tumours is coming close to the end. It comprises 14 volumes, 13 of which are focused on specific organ systems (e. g. digestive tract tumours), topology (e. g. head and neck tumours) or age of incidence (e. g. childhood tumours) [4]. For the first time ever, the fifth edition of the WHO Classification of Tumours contains a separate volume on GTS [1, 2, 4]. During writing of this article, this last issue of the edition is in page-proof stage with a beta version available online [1]. This beta version is the basis of the present review. Though the classification may be subject to minor changes during the editing process, it is unlikely that the taxonomy itself will alter [1]. Note that in addition to this new Blue Book on GTS, the 13 different organ or topology related books also contain separate chapters on genetic tumour (predisposition) syndromes, highlighting the increasing importance of constitutional (germline) genetic alterations for tumour classification [4, 5].

## Hierarchical classification of genetic tumour syndromes according to linnean principles

**Figure 1:**
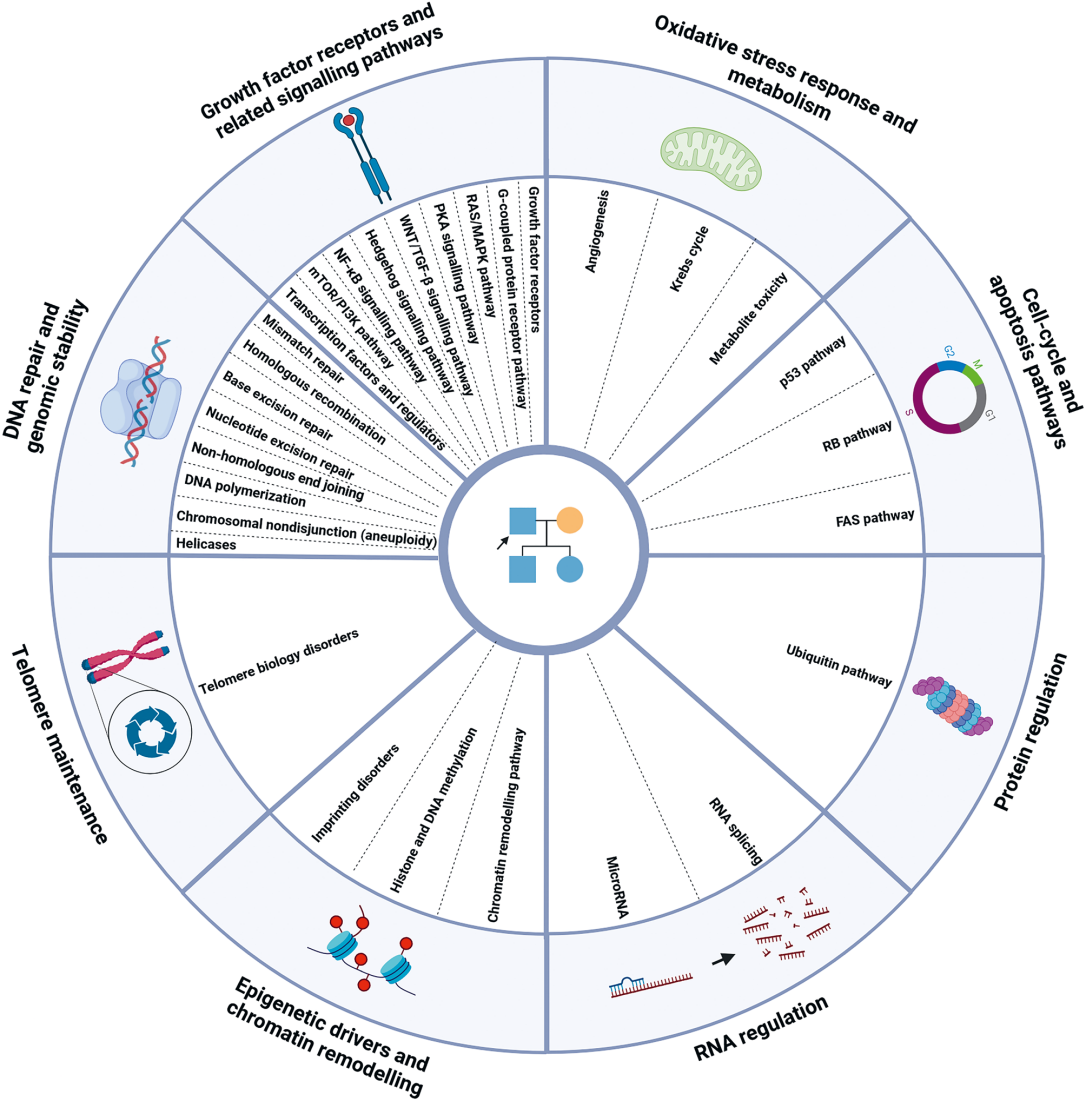
Graphical display of the major mechanism affected and the associated molecular pathways involved in genetic tumour predisposition providing the both upper hierarchical levels for the proposed WHO classification of Genetic Tumour Syndromes [according to 1, 2]. Created in BioRender. Kouroukli, A. (2025) https://BioRender.com/foofang

A common theme of the fifth edition of the WHO Classification of Tumours is that the classification follows a hierarchical structure based on Linnean principles [1, 2, 4]. The process developing this hierarchical system was overseen by a WHO Classification of Tumours Editorial Board for each volume, composed of standing and expert members. In order to develop such a hierarchical system for GTS, the imperfect correlation between diagnosable clinical syndromes and genes posed a considerable challenge. This was largely overcome by a taxonomic organization with four ordinal levels (Figure 1, Table 1) [1, 2]:

Level 1: major (cellular) mechanism affected
Level 2: molecular pathway involved
Level 3: the (clinical) syndrome
Level 4: individual gene(s) affected

In accordance with the different levels, interdisciplinary teams of authors wrote the separate sections detailing information in each GTS including its pattern of tumour localization, clinical features, epidemiology, etiology, pathogenesis, histopathology, diagnostic molecular pathology, staging, and prognosis and prediction [1, 2]. The individual sections were mainly written from the third (syndromic) level, but in few instances also at the fourth level to the level of single genes. The fourth level in many instances is regarded as “subtype”. Important guidance for daily clinical practice is given in the definition of *essential* diagnostic criteria needed to be met for a definite diagnosis, and *desirable* diagnostic criteria, which are important but may or may not be fulfilled [1, 2].

## Terminology and nomenclature 

A variety of terms are in use to describe the conditions detailed in the classification, including expressions like familial/hereditary/genetic and cancer/tumour [2]. The Editorial Board finally applied the term “Genetic Tumour Syndromes” emphasizing the fact that the conditions described need not necessarily be familial or hereditary, and indeed can occur commonly due to *de novo* or post-zygotic pathogenic variants (as also highlighted in the accompanying article on constitutional mosaicism by Boros and colleagues) [2, 6]. The term “tumour”, in contrast e. g. to cancer, refers to the fact, that several of the GTS also include benign neoplastic conditions as part of the phenotype [2].

As the introductory chapters, the Editorial Board also discussed to use the term “constitutional” over “germline” in the context of pathogenic sequence variants for GTS, as these need not be transferred via the germline, but there were also linguistic and historical reasons against using this term [2].

The historically developed naming of GTS does not follow common rules, and eponymous terms exist next to tumour type-centered expressions [2]. As outlined above, in line with the diagnostic approach, the description of the GTS follows the (clinical) syndrome. The WHO classification of GTS undertakes the first (but probably not the last) steps towards a more gene-based nomenclature, e. g. in coining the term “BRCA-related cancer predisposition syndrome”. In particular it follows in many but not all instances recent proposals to systematically name Mendelian disorders. In that regard, the GTS classification paves the way towards a regularized nomenclature as is currently being developed (e. g. by the Mondo Disease Ontology (Mondo)) [7, 8, 9]. It needs to be emphasized, that the term “related” is used instead of “associated” to highlight the direct connection between alteration of the gene and phenotypic manifestation of the syndrome [2, 7]. Nevertheless, taking an overall conservative and pragmatic approach, the WHO Classification nomenclature follows common usage and – considering world-wide applicability – the comparatively easy application of clinical as compared to genetic features for diagnosis [2]. One prominent example for such common and clinically oriented nomenclature is the term Familial Adenomatous Polyposis (FAP), which has not been substituted (yet) by the gene-based term “*APC*-related polyposis” or even “*APC*-related cancer predisposition syndrome”. As usual throughout the fifth edition, the classification also lists alternative terms for several GTS and classifies them as acceptable, or not recommended [1, 2].

## Reporting sequence variants

In line with international standards, the WHO Classification of GTS refers to the international systems to describe genes and variants [10]. In particular, naming of genes follows the rules of the HUGO Gene Nomenclature Committee (HGNC) [11]. To describe genetic alterations, the more neutral term “variant” is used, not “mutation” [10]. In order to adjust for this terminology and to avoid confusion, subsets of tumour entities previously called “variants” are now called “subtypes” [2, 10].

For the description of sequence variants, the HGVS Sequence Variant Nomenclature guidelines is followed [12]. To this end, valid reference sequences should be selected. For genomic alterations, genome build GRCh38 is recommended as reference. For reporting transcriptional variants, the MANE Select transcript for the gene in question should be used [12]. The genome (g.), transcript (c.), and protein (p.) levels should always be applied, with each p. description requiring at least the corresponding c. description [12]. Thus, the WHO Classification of GTS here widely follows human genetics standards.

## What is included – some new kids on the block

We cannot and will not attempt to fully review the content of a several hundred pages book in this article [1]. Therefore, we also refrain from discussing the systematic presentation of the content but instead refer the reader to the tabular display (Table 1) [1, 2].

Nevertheless, we would like to highlight that the WHO Classification of GTS describes several comparatively new syndromes probably not that widely recognized yet. One example is the “Microprocessor-related tumour predisposition syndromes” in the group of GTS associated with the mechanism “RNA regulation” [1, 13]. These are defined as “autosomal dominant tumour predisposition syndromes of uncertain penetrance caused by heterozygous germline pathogenic variants in either of the two genes that together encode the microprocessor (*DROSHA* and *DGCR8*). Variants in *DROSHA* are characterized by the occurrence of pineoblastoma and likely Wilms tumour, whereas a single variant in *DGCR8* leads to thyroid neoplasms and schwannomas” [13, 14, 15]. Another rather recently described GTS is “*ELP1*-related medulloblastoma predisposition syndrome”, which is defined as an “autosomal dominant disorder caused by germline pathogenic sequence variants in the *ELP1* gene and characterized by an increased risk of sonic hedgehog (SHH)-activated medulloblastoma during childhood.” [16]. It belongs to the family of GTS related to alterations in growth factor receptors and related signaling pathways, and affects the sonic hedgehog signaling pathway [1, 16, 17]. A third example is “*MBD4*-related neoplasia syndrome” in the family of base excision repair pathway-associated GTS [18]. This is an autosomal recessive predisposition to various neoplasm including adenomatous intestinal polyposis, colorectal adenocarcinoma, acute myeloid leukaemia, and uveal melanoma. It is related to pathogenic variants affecting the *MBD4* gene encoding Methyl-CpG-Binding Domain Protein 4 [18, 19, 20].

Notably, these GTS discussed above have yet been documented in very few affected persons.

## What is not included – be aware of the gaps

The WHO Classification of GTS does not aim for completeness regarding tumour predisposing genetic conditions. It is expected that the classification will evolve with future editions [2]. Therefore, we want to alert the reader that the upcoming Blue Book on GTS will have some gaps and exclusions due to “limits of time and space that precluded their consideration” [2]. These gaps affect particularly, but not exclusively, the predisposition to leukaemias and lymphomas with several reasons for that [20]. First, the applied pathway-based hierarchical classification leaves few emerging or rare germline tumour syndromes currently unmapped, like the *SAMD9*-related haematological tumour predisposition syndrome (MIRAGE; MIM 617053) [20, 21]. Second, some predispositions do not act “directly” on tumourigenesis but more “indirectly”, most prominently by altering host immunity [20]. Many Inborn Errors of Immunity (IEI) conditions belong to this group of “indirectly acting” genetic tumour predisposing syndromes, for the classification of which it is recommended to apply the regularly updated classification of the International Union of Immunological Societies [20, 22]. This follows a deliberate decision to omit syndromes that cause neoplasia in a secondary manner by altering the immune system or result in tumor-like lesions due to abnormal tissue deposits. Additionally, germline transmission of oncogenic viruses (e. g. HTLV) or germline predisposition to infections related to malignancy (e. g. sickle cell trait/malaria in Burkitt lymphoma) is not covered [22]. Many of these leave-outs affecting predisposition to leukaemias and lymphomas are, as acknowledged in a separate introductory chapter of the WHO Classification of GTS, nevertheless discussed in detail in the fifth edition of the WHO Classification of Haematolymphoid Tumours as outlined previously in this journal [5, 20, 23].

**Table 1:** Overview on the beta-version of the WHO Classification of Genetic Tumour Syndromes (from [1, 2] with few modifications for nomenclature consistency [13])

Mechanism	Molecular Pathway	Genetic Tumour Syndrome*	Gene(s) affected
**Growth factor receptors and related signalling pathways**	**Growth factor receptors**	Hereditary papillary renal carcinoma	*MET*
		Multiple endocrine neoplasia type 2	*RET*
		Juvenile polyposis syndrome	*BMPR1A, SMAD4*
		Hereditary neuroblastoma	*ALK, PHOX2B*
		Encephalocraniocutaneous lipomatosis	*FGFR1*
	**G-coupled protein receptor pathway**	Glucagon cell hyperplasia and neoplasia	*GCGR*
		McCune-Albright syndrome	*GNAS*
		Sturge-Weber syndrome	*GNAQ*
	**RAS/MAPK pathway**	Neurofibromatosis type 1	*NF1*
		NF2-related schwannomatosis	*NF2*
		Costello syndrome	*HRAS*
		Noonan syndrome	various genes
		Schimmelpenning-Feuerstein-Mims	*HRAS, KRAS*
		Gastrointestinal stromal tumour predisposition syndrome	*KIT, PDGFRA*
	**PKA signalling pathway**	Carney complex	*PRKAR1A, PDE8B, PDE11A*
		PIK3CA-related overgrowth spectrum syndromes	*PIK3CA*
	**WNT/TGF-β signalling pathway**	Familial adenomatous polyposis	*APC*
		Gastric adenocarcinoma and proximal polyposis of stomach	*APC* promoter
		AXIN2-related polyposis	*AXIN2*
		Serrated polyposis	*RNF43*
		WT1-related tumour predisposition syndrome	*WT1*
		WAGR syndrome	*WT1*
		Multiple endocrine neoplasia type 1	*MEN1*
		Peutz-Jeghers syndrome	*STK11*
		Hereditary gastric and breast cancer syndrome	*CDH1, CTNNA1*
		Hereditary mixed polyposis syndrome	*GREM1*
	**Hedgehog signalling pathway**	Naevoid basal cell carcinoma syndrome (Gorlin syndrome)	*PTCH1, SUFU, GPR161*
		SMO-related Curry-Jones syndrome	*SMO*
		ELP1-related medulloblastoma predisposition syndrome	*ELP1*
		Osteochondromatosis	*EXT1, EXT2*
	**NF-κB signalling pathway**	Brooke-Spiegler syndrome	*CYLD*
	**mTOR/PI3K pathway**	Tuberous sclerosis	*TSC1, TSC2*
		PTEN hamartoma tumour syndrome	*PTEN*
		Activated PI3K-delta syndrome	*PIK3CD*
	**Transcription factors and regulators**	Multiple endocrine neoplasia type 5, MAX-related tumours	*MAX*
		MAFA-related familial insulinomatosis	*MAFA*
		Birt-Hogg-Dube syndrome	*FLCN*
		Hereditary thrombocytopenia and haematological cancer predisposition syndrome	*RUNX1*
		Familial chordoma	*TBXT*
		Hyperparathyroidism jaw tumour syndrome	*CDC73*
**Oxidative stress response and metabolism**	**Angiogenesis**	Von Hippel-Lindau syndrome	*VHL*
	**Krebs cycle**	SDH-deficient tumour syndromes – hereditary phaeochromocytoma-paraganglioma syndromes	*SDHA, SDHB, SDHC, SDHD, SDHAF2*
		Hereditary leiomyomatosis and renal cell carcinoma syndrome	*FH*
	**Metabolite toxicity**	Hereditary tyrosinaemia type 1	*FAH*
**Cell-cycle and apoptosis pathways**	**p53 pathway**	Li-Fraumeni syndrome	*TP53*
	**RB pathway**	Retinoblastoma syndrome	*RB1*
		Multiple endocrine neoplasia type 4	*CDKN1B*
		CDKN2A-related tumour predisposition syndrome	*CDKN2A*
		CDK4-related melanoma predisposition syndrome	*CDK4*
	**FAS pathway**	Autoimmune lymphoproliferative syndrome	*FAS*
**DNA repair and genomic stability**	**Mismatch repair**	Lynch syndrome	*MLH1, PMS2, MSH2*^1^*, MSH6*
		Muir-Torre syndrome	*MLH1, PMS2, MSH2*^1^*, MSH6*
		Constitutional mismatch repair deficiency syndrome	*MLH1, PMS2, MSH2*^1^*, MSH6*
	**Homologous recombination**	BRCA-related cancer predisposition syndromes	*BRCA1, BRCA2*
		PALB2-related cancer predisposition syndrome	*PALB2*
		RAD51-related cancer predisposition syndromes	*RAD51C, RAD51D*
		Fanconi anaemia	*FANC* genes
	**Base excision repair**	MUTYH-related polyposis	*MUTYH*
		NTHL1-related tumour syndrome	*NTHL1*
		MBD4-related neoplasia syndrome	*MBD4*
	**Nucleotide excision repair**	Xeroderma pigmentosum	various genes
	**Non-homologous end joining**	Ataxia-telangiectasia	*ATM*
		CHEK2-related hereditary (breast) cancer predisposition syndrome	*CHEK2*
		Nijmegen breakage syndrome	*NBN*
	**DNA polymerization**	Polymerase proofreading-related polyposis	*POLD1, POLE*
	**Helicases**	Bloom syndrome	*BLM*
		Werner syndrome	*WRN*
		Rothmund-Thomson syndrome	*ANAPC1, RECQL4*
		DDX41-related haematological tumour predisposition syndrome	*DDX41*
	**Chromosomal nondisjunction (aneuploidy)**	Mosaic variegated aneuploidy	*BUB1B, CEP57*^2^*, TRIP13, BUB1, BUB3*
		Klinefelter syndrome	* *
		Turner syndrome	* *
		Down syndrome	* *
**Telomere maintenance**	**Telomere biology disorders**	Dyskeratosis congenita	*DKC1, TERT, TERC, TINF2,* other* IBMFS* genes
		POT1- and shelterin-related tumour predisposition syndrome	*POT1, ACD, TERF2IP, TERT* promoter
**Epigenetic drivers and chromatin remodelling**	**Imprinting disorders**	Beckwith-Wiedemann spectrum	*IGF2, CDKN1C*
	**Histone and DNA methylation**	Enchondromatosis	*IDH1, IDH2*
	**Chromatin remodelling pathway**	Rhabdoid tumour predisposition syndrome	*SMARCB1, SMARCA4*
		Schwannomatosis	*SMARCB1, LZTR1*
		Clear cell meningioma predisposition syndrome	*SMARCE1*
		Weaver syndrome	*EZH2*
**RNA regulation**	**MicroRNA**	DICER1-related tumour predisposition syndrome	*DICER1*
		Microprocessor-related tumour predisposition syndrome	*DROSHA, DGCR8*
	**RNA splicing**	Goldenhar syndrome	*MYT1, SF3B2*
**Protein regulation**	**Ubiquitin pathway**	BAP1-related tumour predisposition syndrome	*BAP1*

*Few names slightly modified from beta-version for consistency (changing “associated” to “related”).

^1^ includes *EPCAM*

^2^ proof of a direct link between CEP57 alteration and predisposition to cancer is pending

## Conclusion and outlook

The fifth edition of the WHO Classification of Tumours includes for the first time, a taxonomy for Genetic Tumour Syndromes, which sets international standards for disease definition, diagnosis and nomenclature [1]. Under the guidance of the International Agency for Research on Cancer, the proposed classification has been conceptualized, developed and specified by numerous experts in the field acting as members of the editorial board or authors with world-wide coverage [2]. Thus, as holds true also for other volumes of the WHO Classification of Tumours, wide acceptance of the classification e. g. by regulatory bodies throughout the world is expected. Whereas the present review is based on the online beta-version of the classification [1], the final version will be available in form of a printed “Blue Book” as well as in a searchable database format that is also accessible online (https://tumourclassification.iarc.who.int) [24]. The latter will also be helpful to implement regular (planned 5-yearly) updates and revisions in accordance with scientific advances, which likely will also address present gaps in the systematics.
